# AraC interacts with p75^NTR^ transmembrane domain to induce cell death of mature neurons

**DOI:** 10.1038/s41419-023-05979-7

**Published:** 2023-07-17

**Authors:** Vanessa Lopes-Rodrigues, Pia Boxy, Eunice Sim, Dong Ik Park, Michael Habeck, Josep Carbonell, Annika Andersson, Diana Fernández-Suárez, Poul Nissen, Anders Nykjær, Lilian Kisiswa

**Affiliations:** 1 Department of Physiology and Life Sciences Institute, National University of Singapore, Singapore, 117597 Singapore; 2 Department of Biomedicine, Aarhus University, Aarhus, Denmark; 3 Danish Research Institute of Translational Neuroscience (DANDRITE)–Nordic EMBL Partnership for Molecular Medicine, Aarhus University, Aarhus, Denmark; 4 The Danish National Research Foundation Center, PROMEMO, Aarhus University, Aarhus, Denmark; 5 Department of Molecular Biology and Genetics, Aarhus University, Aarhus, Denmark; 6 Department of Neuroscience, Karolinska Institute, Stockholm, S-17177 Sweden

**Keywords:** Cell death in the nervous system, Cell death

## Abstract

Cytosine arabinoside (AraC) is one of the main therapeutic treatments for several types of cancer, including acute myeloid leukaemia. However, after a high-dose AraC chemotherapy regime, patients develop severe neurotoxicity and cell death in the central nervous system leading to cerebellar ataxia, dysarthria, nystagmus, somnolence and drowsiness. AraC induces apoptosis in dividing cells. However, the mechanism by which it leads to neurite degeneration and cell death in mature neurons remains unclear. We hypothesise that the upregulation of the death receptor p75^NTR^ is responsible for AraC-mediated neurodegeneration and cell death in leukaemia patients undergoing AraC treatment. To determine the role of AraC-p75^NTR^ signalling in the cell death of mature neurons, we used mature cerebellar granule neurons’ primary cultures from p75^NTR^ knockout and *p75*^*NTRCys259*^ mice. Evaluation of neurite degeneration, cell death and p75^NTR^ signalling was done by immunohistochemistry and immunoblotting. To assess the interaction between AraC and p75^NTR^, we performed cellular thermal shift and AraTM assays as well as Homo-FRET anisotropy imaging. We show that AraC induces neurite degeneration and programmed cell death of mature cerebellar granule neurons in a p75^NTR^-dependent manner. Mechanistically, Proline 252 and Cysteine 256 residues facilitate AraC interaction with the transmembrane domain of p75^NTR^ resulting in uncoupling of p75^NTR^ from the NFκB survival pathway. This, in turn, exacerbates the activation of the cell death/JNK pathway by recruitment of TRAF6 to p75^NTR^. Our findings identify p75^NTR^ as a novel molecular target to develop treatments for counteract AraC-mediated cell death of mature neurons.

## Background

AraC (1-β-arabinofuranosylcytosine or cytosine arabinoside) is the most effective chemotherapy agent used to treat patients with acute myeloid leukaemia (AML), as well as other types of haematological cancers [1–5]. In order to achieve the therapeutic efficacy of AraC, patients are subjected to a high-dose AraC (HIDAC) chemotherapy regime [1]. Although HIDAC is efficient in treating AML, it leads to severe cerebellar neurotoxicity [6, 7]. It has been suggested that AraC elicits neurotoxicity by inducing programmed cell death in cerebellar neurons, in particular, cerebellar granule neurons (CGNs) [8, 9]. However, this has only been shown for immature CGNs during development [8, 9]. Noteworthy, AraC-mediated neurotoxicity is prevalent in patients over 50 years old as they are more likely to receive HIDAC [3, 10, 11]. Generally, neuronal proliferation ceases after the developmental period, with the exception of a few brain regions, such as the hippocampus and olfactory bulb [12, 13]. AraC has been shown to induce apoptosis in proliferating cells by inhibiting DNA synthesis [14, 15] and DNA repair [16]. Therefore, it is unlikely that the mechanism by which AraC induces cerebellar neurotoxicity in adult patients is by targeting proliferating neurons. This warrants the elucidation of the mechanisms by which AraC induces cell death of mature neurons.

The death receptor p75^NTR^ is highly expressed in the nervous system during development, but it is widely downregulated in the adult brain [17], with the exception of the cholinergic neurons in the basal forebrain [18]. However, low expression of p75^NTR^ persists into adulthood in some areas of the central nervous system (CNS), such as the cerebellum, septum, medulla and pons [19]. Moreover, a growing body of evidence has demonstrated an upregulation of p75^NTR^ under pathological conditions, including cancer, brain injury and neurodegenerative diseases [20–23]. Interestingly, an increase in p75^NTR^ expression in the serum and peripheral blood of leukaemia patients has been reported [24, 25]. Therefore, we asked whether p75^NTR^ could be responsible for mediating AraC-induced neurite degeneration and cell death of mature cerebellar neurons. Here, we show that AraC induces neurite degeneration and apoptosis of mature CGNs by interacting with the transmembrane domain (TMD) of p75^NTR^, an interaction that is dependent on the Proline (Pro) 253 and Cysteine (Cys) 256 residues. Functionally, we show that AraC interaction with the p75^NTR^ TMD uncouples p75^NTR^ from the NFkB survival pathway, resulting in the exacerbated activation of the cell death/JNK pathway in CGNs.

## Materials and methods

### Animals

Mice were housed in a 12-hour light/dark cycle and fed a standard chow diet. The mutant mouse lines used were p75^NTR^ knockout (*p75*^*NTR−/*−^) [26] and *p75*^*NTRCys259*^ (knock-in mice carrying a substitution of cysteine at position 259 to alanine) [27] mice. Both transgenic mouse lines were maintained on a C57BL/6J background. Mice of both sexes were used for the experiments. All animal experiments were conducted in accordance with the National University of Singapore Institutional Animal Care, Use Committee and the Stockholm North Ethical Committee for Animal Research regulations and the Danish Animal Experiment Inspectorate Under the Ministry of Justice.

### Neuronal cultures

P7 mouse cerebella were dissected with the removal of the meninges in ice-cold phosphate saline buffer (PBS). Whole cerebella were then digested with TrypLE™ Express (Gibco, 12604021) for CGNs extraction. CGNs were plated at a density of 40,000 cells per coverslip coated with poly-d-lysine (Sigma, P1524) in a 24-well plate (Thermo Scientific, 142475) in neurobasal medium (Gibco, 21103049) supplemented with 25 mM KCl, 1 mM Glutamax (Gibco, 35050061), 1× Pen/Strep (Sigma, P4333), 10 μM AraC (Sigma, C-6645), 1× B27 supplement (Gibco, 17504044). AraC was diluted in PBS and used at a concentration of 10 μM for the elimination of glia cells in the neuronal cultures. For experimental conditions, AraC was used at concentrations of 500 and 1000 μM. These high concentrations were used to reflect the high doses (2–3 g/m^2^) of AraC per dose given to patients on a HIDAC chemotherapy regime [28–30]. HIDAC protocol (2–3 g/m^2^ every 12 h for up to 6 doses per round of treatment) has been shown to result in AraC concentration exceeding 100 μM in plasma [31].

### Neurite degeneration

To assess neurite degeneration, 4 days in vitro (DIV) wild-type neurons were treated for 24 or 48 h with 500 μM AraC. After treatment, CGNs were fixed for 10 min with ice-cold methanol. Cells were then permeabilized and blocked in 5% normal donkey serum (Jackson ImmunoResearch: 017-000-121) and 0.3% Triton X-100 (Thermo Scientific; 85111) in PBS. Cells were incubated overnight at 4 °C with mouse anti-β-III tubulin (R&D Systems, MAB1195; 1:2500) and counterstained the following day with donkey anti-mouse Alexa Fluor 488 (Abcam, ab150105; 1:2000) and Hoechst (Sigma, B2261; 1:2000). Images were taken using a Confocal Microscope LSM 780 Zeiss Axio Observer fluorescence microscope. Images were captured from regions with well-separated neurites. NIH ImageJ software was used to threshold and binarize the images and remove all cell bodies, after which the Analyse Particles algorithm was applied to identify the area of fragments based on size (20–10,000 pixels) and circularity (0.2–1.0). The degeneration index (DI) was then calculated as the ratio of the total area of detected neurite fragments over the total neurite area. In agreement with previous studies [32], a DI of 0.2 or higher indicated neurite degeneration.

### Cell death

Apoptosis was assessed in WT, *p75*^*NTR−/−*^ and *p75*^*NTRCys259*^ CGNs treated for 24 h with either 500 μM or 1000 μM AraC starting at 4 DIV. Apoptotic cells were labelled using Click-iT plus TUNEL assay for in situ apoptosis detection kit (Thermo Scientific, Cat: C10617) according to manufacturer instructions. Neurons were also stained for cleaved caspase 3 (Cell Signalling Technology, 9761, 1:400), β-III tubulin and DAPI (Sigma; D9542; 1:10,000) following the protocol for immunocytochemistry explained below. For each experiment and treatment, neurons were cultured in duplicates, and at least 15 images were taken per coverslip with a Zeiss Axioplan confocal microscope. The number of cells positive for cleaved caspase 3 and TUNEL was quantified using NIH ImageJ software.

### Protein collection and immunoblotting

To collect protein for immunoblotting, WT neurons were cultured at high density (~200,000 neurons per well) in a 48-well plate. Four days after plating, cells were stimulated with 500 μM AraC for 15, 30 and 60 min.

Protein samples were prepared for SDS-PAGE in SDS sample buffer (Merck Millipore; 70607) and boiled at 95 °C for 10 min before electrophoresis on 12% gels. Proteins were transferred to PVDF membranes (Amersham, GE10600023). Membranes were blocked with 5% non-fat milk and incubated with primary antibodies.

The following primary antibodies were used at the indicated dilutions: rabbit anti-phospho Y515 TrkB (Abcam: ab131483; 1:500), goat anti-TrkB (R&D systems; AF1494; 1:500), rabbit anti-IκBα (Santa Cruz; 9165; 1:500), rabbit anti-phospho-c-Jun (Thr91, Cell Signalling Technology; 2303; 1:1000), rabbit anti-c-Jun (Cell Signalling Technology; 9165; 1:1000) and mouse anti-GAPDH (Sigma; G8795; 1:1000). Immunoreactivity was visualised using appropriate HRP-conjugated secondary antibodies (Cell Signalling Technology; 7074). Immunoblots were developed using the ECL Advance Western blotting detection kit (Thermo Scientific; 34095) and imaged using a chemiluminescent western blot imaging system, Azure c300 (Azure Biosystems). Image analysis and quantification of band intensities were done using NIH ImageJ software.

### RhoA assay

Protein was extracted from WT CGNs that were treated at 4 DIV with 500 μM AraC for 30 min. RhoA activity was evaluated in total CGNs extracts using the RhoA G-Lisa kit (Cytoskeleton; BK124) following the manufacturer’s instructions. An equal amount of protein was used from each sample as determined by the BCA protein Assay (ThermoFisher Scientific; 23235).

### Proximity ligation assay (PLA)

Four DIV WT CGNs were treated with 500 μM AraC for 10 min. After treatment, CGNs were fixed for 15 min in 4% paraformaldehyde (PFA)/4% sucrose, permeabilized, and blocked in 10% normal donkey serum and 0.3% Triton X-100 in PBS for 1 h. Neurons were then incubated overnight at 4 °C with anti-p75^NTR^ (Promega; G323A; 1:500) and anti-TRAF6 (Santa Cruz; sc-8490; 1:100) antibodies in PBS supplemented with 3% BSA. The Duolink In Situ Proximity Ligation kit (Sigma; DUO92007) was used as per the manufacturer’s instructions. Cells were imaged with an LSM Imager Z2 confocal microscope (Zeiss) to detect PLA signals. PLA puncta were quantified using NIH ImageJ software with the plugin particle analyser.

### Immunocytochemistry

For immunocytochemistry, the cultures were fixed in 4% PFA/4% sucrose for 15 min and washed with PBS before blocking nonspecific binding and permeabilizing with blocking solution (5% donkey serum and 0.3% Triton X-100 in PBS) for 1 h at room temperature. Neurons were incubated overnight with the primary antibodies in diluted blocking solution to 1% donkey serum at 4 °C. After washing with PBS, the neurons were incubated with the appropriate secondary antibodies.

The primary antibodies used in this study were: polyclonal anti-cleaved caspase 3 (Cell Signalling Technology; 9761; 1:400), monoclonal anti–β-III tubulin (R&D systems, Cat: MAB1195, 1:10000), polyclonal anti-p75^NTR^ (Neuromics; GT15057; 1:500), polyclonal anti-TrkB (R&D systems; AF1494; 1:250), polyclonal anti-MAP2 (Abcam; ab5392; 1:2000) and polyclonal anti-P65NFkB (Santa Cruz; sc-372; 1:250).

Secondary antibodies were Alexa Fluor–conjugated anti-immunoglobulin from Life Technologies and Invitrogen, used at 1:1500 ((donkey anti-rabbit IgG Alexa Fluor 555, (A31572), donkey anti-mouse IgG Alexa Fluor 488 (A21202), donkey anti-mouse IgG Alexa Fluor 555 (A31570), donkey anti-goat IgG Alexa Fluor 488 (A11055), donkey anti-rabbit IgG Alexa Fluor 488 (A32790), donkey anti-chicken IgG Alexa Fluor 647 (703-496-155). Images were obtained using a Zeiss Axioplan confocal microscope. The number of cells positive for cleaved caspase 3 and TUNEL were quantified using NIH ImageJ software.

### Docking analysis

The nuclear magnetic resonance (NMR) structure of the p75^NTR^ TMD dimer in complex with NSC49652 was used for docking of AraC (pdb:5zgg) [33]. NSC49652 was removed, and AutoDock Tools [34] was used for assigning Gasteiger charges and hydrogens to AraC (pbd ligand: AR3). A grid box was centred around the TMD interface, and docking was performed using AutoDock Vina [35]. Proteins*Plus* [36], PoseView [37] and ChimeraX [38] were used for visualisation of 2D interaction diagrams.

### AraTM assay

AraTM assay was used to assess conformation changes and binding strength in a pair interaction of TMDs [39, 40], as previously described [33]. Briefly, TMD cDNAs of the human p75^NTR^ (NLIPVYCSILAAVVVGLVAYIAFKRW) and TrkB (SVYAVVVIASVVGFCLLVMLFLL) were subcloned into AraTM chimera plasmid in between the KpnI and SacI restriction sites. AS19 LPS-negative *Escherichia coli* cells were transformed with the above-mentioned plasmids together with a GFP reporter plasmid. The selected colonies were grown overnight for 18 h in a shaker at 37^o^C in Lysogeny Broth (LB) supplemented with 50 μg/ml Spectinomycin and 100 μg/ml ampicillin. The culture was then diluted 1:100 in fresh LB medium and allowed to grow till optical density (OD) 630 reached between 0.2 and 0.5, after which 1 mM IPTG was added to induce the expression of the p75^NTR^ TMD–AraTM chimaera or TrkB TMD-AraTM chimaera. 100 μl of the culture per well was dispensed in black-rim clear bottom 96-well plates (Corning, cat: 3631) previously plated with serial concentrations (30, 100, 300, 500 and 100 μM) of AraC. The plates were then incubated with vigorous shaking at 38^0^C for 4 h to allow IPTG-induced expression of the TM-AraTM chimaera. The plates were then centrifuged at 4000 rpm for 10 min at room temperature to pellet bacteria. LB media was aspirated and replaced with 100 μl of PBS, and bacteria cells were resuspended by vigorous shaking for 10 min. GFP signal was measured in each well (excitation 475 nm, emission 509 nm), and bacterial density was determined by measurement of OD_630_ in a microplate plate reader (BioTek).

### Cellular thermal shift assay

Cellular thermal shift assay (CETSA) was performed as previously described [41]. 293 T HEK cells constitutively expressing p75^NTR^ protein were homogenised in a buffer containing 100 mM HEPES, 1 mM DTT, 10 mM MgCl_2_, protease inhibitor cocktail tablets (Roche; 11836153001) and phosphatase inhibitors (Roche; 04906837001). After 3 freeze-thaw cycles using liquid nitrogen, the lysate was centrifuged (20,000*g*, 4 °C, 20 min) to collect the supernatant. Protein concentration was measured using BCA assay. The same amount of protein lysate was aliquoted into different PCR tubes and incubated with either PBS (vehicle) or 500 μM AraC for 3 min at room temperature. The samples were then simultaneously subjected to 6 different temperatures (37, 41, 45, 49, 53 and 57 °C) for 3 min in the Veriti Thermal Cycler (Applied Biosystems). After 3 min cooling on ice, heat-treated protein lysate was centrifuged (20,000*g*, 4 °C, 20 min). The supernatant was collected into new tubes, and samples were subjected to immunoblotting. Primary antibodies used were anti-p75^NTR^ (Promega, Cat: G323A, 1:300). The densitometry was done on the immunoblot bands, and restricted cubic spline curve fitting was generated using GraphPad Prism 9 (GraphPad Software, Inc., La Jolla, CA, USA).

### Isothermal dose response-CETSA (ITDR-CETSA)

The protein lysate from 293 T HEK cells constitutively expressing p75^NTR^ protein was homogenised in the same buffer as described above for CETSA and incubated with different concentrations of AraC (0, 0.1, 1, 30, 100, 300, 500 and 1000 μM) for 3 min at RT. The protein extracts were heated at a constant temperature of 37 or 53 °C for 3 min. After subsequent cooling on ice for 3 min, the heat-treated lysate was centrifuged (20,000*g*, 4 °C, 20 min), followed by supernatant collection. Samples were then subjected to immunoblotting and probed for anti-p75^NTR^ (Promega, Cat: G323A, 1:300) and anti-GAPDH (Sigma, Cat: G8795, 1:1000). Densitometry data acquired from 37 °C ITDR-CETSA were used as non-denaturing controls to normalise those from ITDR-CETSA conducted at 53 °C. The densitometry was done on the immunoblot bands, and restricted cubic spline curve fitting was generated using GraphPad Prism 9 (GraphPad Software, Inc., La Jolla, CA, USA).

### Homo-FRET anisotropy imaging

COS-7 cells were cultured under standard conditions in Dulbecco’s modified eagle medium (DMEM)-supplemented with 10% foetal bovine serum, 100 units/ml penicillin, 100 mg/ml streptomycin and 2.5 mM glutamate. Cells were transiently transfected with a rat p75^NTR^-EGFP* fusion constructs [42] using FuGENE transfection reagents (Fisher Scientific, Cat: PRE2311). EGFP* consist of a monomeric A207K EGFP mutant. 24 h later, anisotropy imaging was done as previously described [27, 33, 43]. Changes in anisotropy were expressed as fold change at each time point in comparison to the mean of 6-time points obtained prior to the addition of the vehicle or 500 μM AraC. Images were acquired using Nikon Ti–E-based live cell epi-fluorescence microscope and MetaMorph software and analysed using MatLab from Mathworks.

### Statistical analysis

Data are expressed as mean and standard errors of the mean. No statistical methods were used to predetermine sample sizes, but our sample sizes are similar to those generally used in the field. No data were excluded from the analysis. Following the normality test and homogeneity variance (*F*-test or Kolmogorov–Smirnov test with Dallal–Wilkinson–Lilliefor *P* value), group comparison was made using an unpaired student *t*-test, one-way or two-way ANOVA as appropriate followed by Bonferroni post hoc test for normally distributed data. Differences were considered significant for *P* < 0.05. The experiments were not randomised.

## Results

### AraC induces neurite degeneration and apoptosis in mature CGNs

**Fig. 1:**
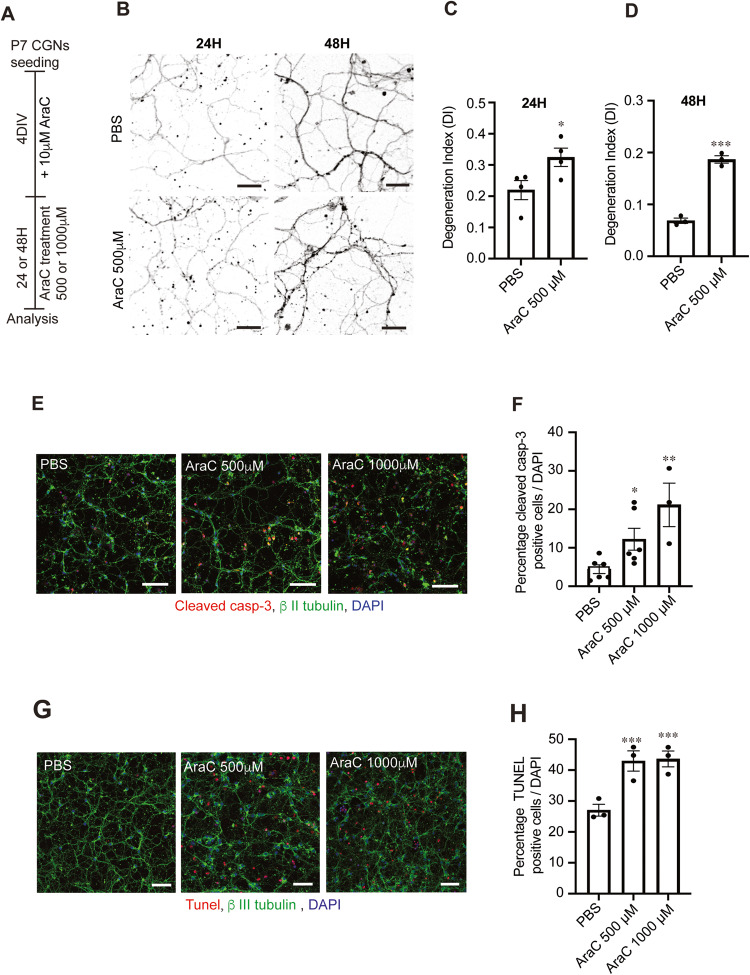
AraC induces neurite degeneration and apoptosis in mature CGNs. **A** A schematic drawing of the cell culture procedure. **B** Image of representative wild-type P7 CGNs neurite grown for 4 DIV that were treated with either PBS (control) or 500 μM AraC for 24 or 48 h and stained for β III tubulin Scale bars, 50 μm. **C**, **D** Quantification of degeneration index in CGNs treated with either PBS (control) or 500 μM AraC for 24 h (**C**) and 48 h (**D**) CGNs cultures (total of 25 images per condition were quantified). Mean ± s.e.m. of data from 3 to 4 separate cultures (*P < 0.05 and ***P < 0.001 compared to control, Unpaired Student *t*-test) is shown. **E** Image of representative P7 CGNs cultured for 4 DIV that were treated with either PBS (control) or 500 μM AraC for 24 h and stained for cleaved caspase 3 (red), anti-β III tubulin (green) and counterstained with DAPI (blue). Scale bars, 50 μm. **F** Quantification of percentage cleaved caspase 3 positive neurons in CGNs treated with PBS (control), 500 μM or 1000 μM AraC for 24 h (a total of 80 images per condition were counted). Mean ± s.e.m. of data from four separate cultures (*P < 0.05 and **P < 0.01 compared to control, one-way ANOVA followed by Bonferroni post hoc test) is shown. **G** Photomicrographs of representative wild-type P7 CGNs cultured for 4 DIV that were treated with either untreated PBS (control), 500 μM or 1000 μM AraC for 24 h and stained for TUNEL (green), with anti-β III tubulin (red) and counterstained with DAPI (blue). Scale bars, 50 μm. **H** Quantification of percentage TUNEL-positive neurons in CGNs treated with PBS, 500 μM or 1000 μM AraC for 24 h (a total of 60 images per condition were counted). Mean ± s.e.m. of data from three separate cultures, ***P < 0.001 compared to control, one-way ANOVA followed by Bonferroni post hoc test) is shown.

Apoptosis of immature CGNs associated with AraC is well-documented [8, 9, 44–46]. However, it is known that adult cancer patients under AraC medication also develop cerebellar neurotoxicity resulting in neurite degeneration and cell death [6, 7] despite a lack of neurogenesis and proliferation in the adult cerebellum. First, we aimed to confirm that AraC induces degeneration and apoptosis in mature CGNs. To obtain mature neurons for the experiments, we performed P7 cerebellar cultures, initially containing a mixture of glial cells and immature CGNs. To eliminate the proliferating glial cells from the culture, we cultured the cells for 4 days in media containing a low concentration of AraC (10 μM: Fig. 1A), obtaining enriched CGN cultures deprived of glial cells (Supplementary Fig. 1A, B). Previous reports suggested that CGN cultures after 4 DIV contain mature-like neurons that present high expression of mature markers such as MEF2 and Zic2 and low levels of immature markers such as Math1 or TAG1 [47]. We confirmed the maturity of our CGNs by staining for TAG1 (Supplementary Fig. 1C). On the 4 DIV, neurons were treated with either PBS (control) or 500 μM AraC for 24 or 48 h (Fig. 1A). Quantification of the neurite DI showed an increase in neurite degeneration in neurons treated with 500 μM AraC for 24 h compared to untreated neurons (Fig. 1B, C). Longer (48 h) exposure of neurons to 500 μM AraC worsened neurite degeneration (Fig. 1B, D). Moreover, cells treated with either 500 μM or 1000 μM AraC for 24 h showed an increase in the number of cleaved caspase 3 positive neurons (Fig. 1E, F). In agreement with this, analysis of the number of TUNEL analyses showed an approximately 2-fold increase in apoptotic activity in CGNs after either 500 μM or 1000 μM AraC-treatment (Fig. 1G, H), suggesting that the lower concentration is sufficient for reaching the plateau on the apoptotic effect. Therefore, these results are in agreement with previous studies [8, 9, 45], indicating that AraC induces neurite degeneration and cell death of mature CGNs

### p75^NTR^ death receptor is required for AraC-induced neurodegeneration and apoptosis in mature CGNs

We and others have shown that the p75^NTR^ death receptor plays an important role in CGNs apoptosis during development [48–52]. Although p75^NTR^ is abundantly expressed in several types of developing neurons, its expression is negligible in the majority of mature neurons [19, 53].

**Fig. 2:**
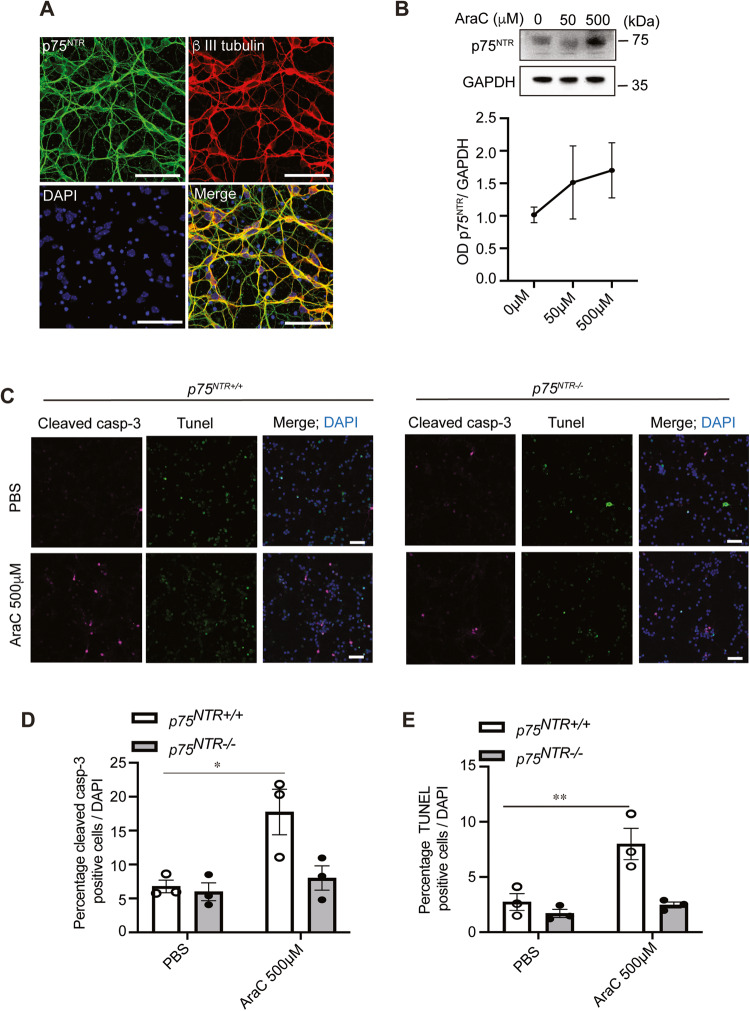
AraC requires p75^NTR^ to induce apoptosis in mature CGNs. **A** Representative micrographs of wild-type P7 CGNs cultured for 4 DIV and double stained with anti-p75^NTR^ together with anti- β III tubulin and counterstained with DAPI. Scale bars, 50 μm. **B** Representative and quantification of immunoblots showing the expression of p75^NTR^ in 4DIV CGNs treated with PBS (0 μM AraC), 50 μM or 500 μM AraC for 24 h. **C** Image of representative P7 *p75*^*NTR+/+*^ *and p75*^*NTR−/−*^ CGNs cultured for 4 DIV, treated with either PBS (control) or 500 μM AraC for 24 h and stained for anti-cleaved caspase 3 (magenta), TUNEL (green) and counterstained with DAPI (blue). Scale bars, 50 μm. **D**, **E** Quantification of percentage cleaved caspase 3 positive (**D**) and TUNEL positive (**E**) in *p75*^*NTR+/+*^ *and p75*^*NTR−/−*^ neurons treated with PBS or AraC (500 μM or 1000 μM for 24 h (total of 80 images per condition were counted)). Mean ± s.e.m. of data from four separate cultures, *P < 0.05 and **P < 0.01 compared to control, one-way ANOVA followed by Bonferroni post hoc test) is shown.

However, some areas of the adult brain, such as the cerebellum, contain low levels of p75^NTR^. Interestingly, it has also been observed that upon injury and neurodegeneration, p75^NTR^ expression is upregulated in the CNS [20, 22, 54]. We, therefore, asked whether the cerebellum is especially vulnerable to AraC-induced cell death due to the presence of p75^NTR^ in mature cerebellar neurons. First, we confirmed the expression of p75^NTR^ in mature CGNs (Fig. 2A). Next, we assessed whether the addition of AraC to these neurons could trigger the expression of p75^NTR^, therefore increasing their vulnerability to AraC-induced cell death. We found that increasing doses of AraC increased the expression of p75^NTR^ in cultured CGNs (Fig. 2B). Then, we tested whether AraC-mediated neuronal death requires p75^NTR^. Wild type (*p75*^*NTR+/+*^) and p75^NTR^ knockout (*p75*^*NTR−/−*^) CGNs were treated at 4 DIV with PBS (control) or AraC for 24 h, and then apoptotic activity was evaluated using cleaved caspase 3 and TUNEL assays. As expected, the number of WT CGNs positive for cleaved caspase 3 (Fig. 2C, D) and TUNEL (Fig. 2C, E) increased 2-fold after AraC treatment. *p75*^*NTR−/−*^ neurons treated with AraC did not show an increase in apoptotic activity (Fig. 2C–E). Altogether, these data suggest that AraC-mediated neuronal death requires p75^NTR^ death receptor.

### AraC interacts with p75^NTR^

**Fig. 3:**
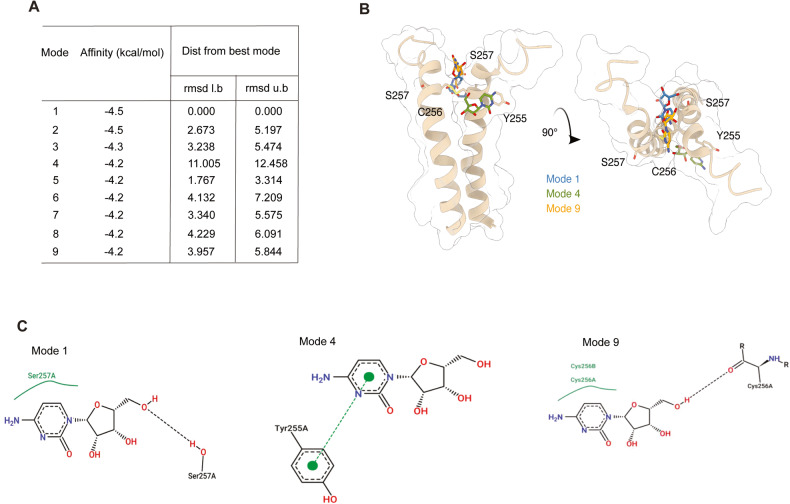
Molecular docking results of AraC and p75^NTR^ TMD interaction. **A** Ranked list of potential AraC binding sites and their distance from the highest scoring model. **B** Three principal AraC binding sites were identified and are shown in blue, green and orange stick representation docked onto p75^NTR^ TMD. p75^NTR^ TMD is shown in tan with a transparent surface, and residues potentially interacting with docked AraC are shown in stick representation. **C** 2D interaction diagrams of the three respective modes.

Although our data show that in the absence of p75^NTR^, AraC is unable to induce apoptosis in mature neurons, it remains unclear whether this effect was a result of a direct interaction between AraC and p75^NTR^ or an indirect effect through p75^NTR^ interacting partners. To assess this, we performed an in silico analysis to predict the possible interaction of AraC to p75^NTR^. We recently reported that a small molecule (NSC49652) binds to the p75^NTR^ TMD and induces cell death both in neurons and cancer cells [33]. Therefore, we speculated that AraC might interact with the TMD of p75^NTR^. Using the p75^NTR^ TMD structure that we previously reported [33], our molecular docking data (Fig. 3) suggest that AraC potentially binds to p75^NTR^ TMD in a similar region as NSC49652 [33].

**Fig. 4:**
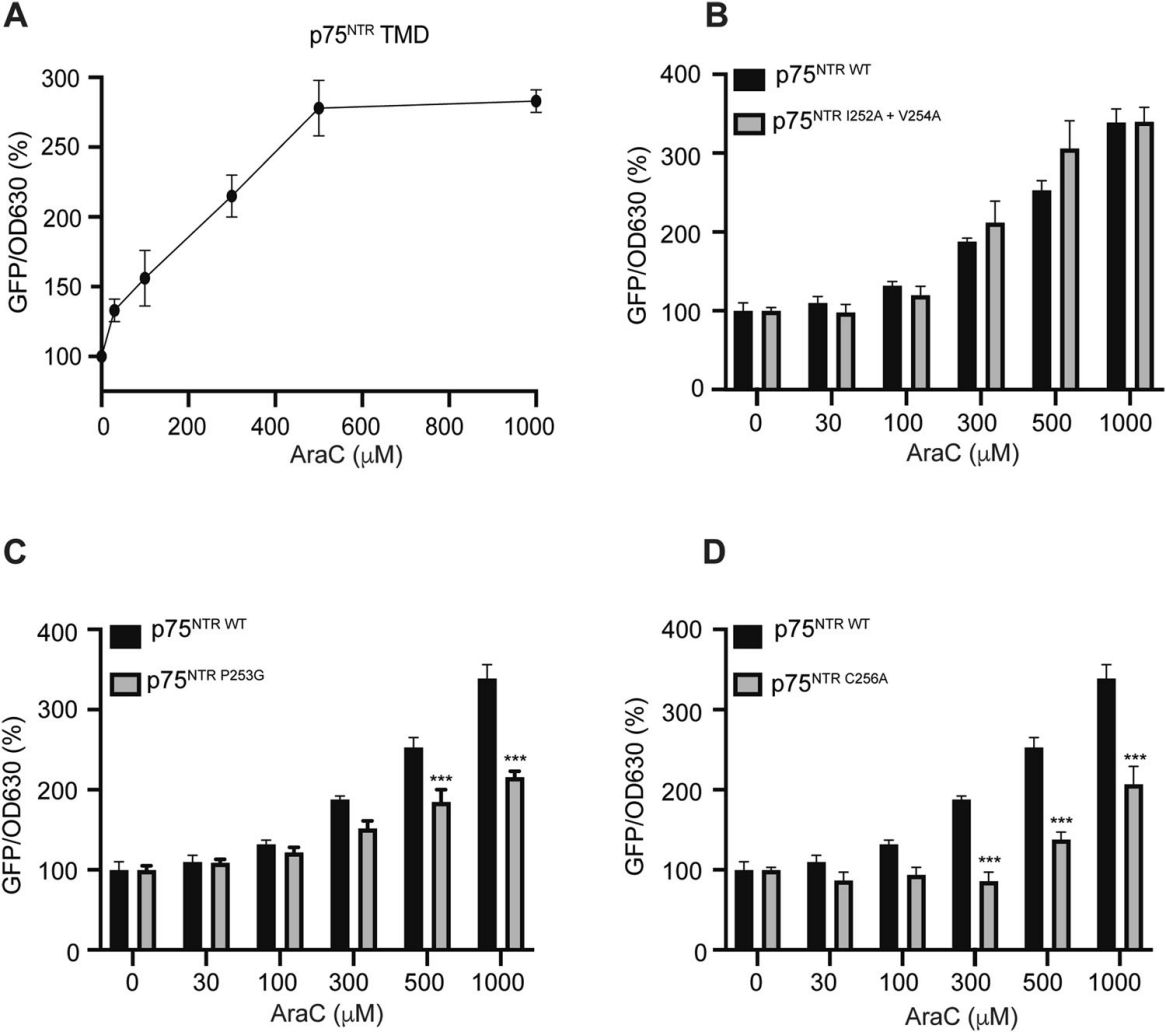
AraC requires Pro253 and Cys256 to interact with p75^NTR^ TMD. **A** Dose response of AraC in the AraTM assay of p75^NTR^. Results are plotted as means ± SD (*N* = 3). **B**–**D** Comparison of human wild-type p75^NTR^ TMD with p75^NTR^ TMD with I252A plus V254A (**B**) or P253G (**C**) or C256A (**D**) mutants in the AraTM assay in response to increasing doses of AraC. The GFP over OD630 signal without any drug added was set at 100%. Results are plotted as means ± SD (*N* = 3). Significance was calculated using the two-way ANOVA followed by Bonferroni post hoc test where **p < 0.01 and ***p < 0.001.

Next, we evaluated whether AraC could bind to the TMD of p75^NTR^ using an AraTM assay where the bacteria expressed only the TMD of p75^NTR^. To assess the specificity of AraC binding to p75^NTR^ TMD, we used TMD from a different receptor, namely TrkB (Supplementary Fig. 2B). We found that AraC preferentially binds with p75^NTR^ TMD (Fig. 4A) although there was some minor binding to TrkB TMD (Supplementary Fig. 2B). After confirming the interaction of AraC to the p75^NTR^ TMD, we sought to determine which residues facilitated this interaction. The residues of human p75^NTR^ TMD are as follows NLIPVYCSILAAVVVGLVAYIAFKRW, starting at residue 250. Using a plasmid carrying human p75^NTR^ TMD, we mutated isoleucine 252 and valine 254 residues to alanine (I252A and V254A, respectively), located at the beginning of p75^NTR^ TMD. Both bacteria transfected with human wild-type p75^NTR^ TMD and p75^NTR^ TMD carrying the double mutants, I252A and V254A, responded similarly to AraC treatment (Fig. 4B), suggesting that these two residues are not required for the AraC/p75^NTR^ interaction. We then tested human p75^NTR^ TMD carrying mutation P253G, where Pro 253 was replaced with glycine. Upon AraC treatment, the bacteria carrying the P253G mutation had a lower percentage of GFP/OD630 compared to bacteria carrying wild-type p75^NTR^ TMD (Fig. 4C). This result suggests that the Pro 253 is required for AraC binding to p75^NTR^ TMD. We then tested another point mutation, in which the Cys 256 was replaced with alanine (C256A mutant). Similar to P253G, the C256A mutation had a lower percentage of GFP/OD630 compared to wild-type p75^NTR^ TMD upon AraC treatment (Fig. 4D). Interestingly, the response of the C256A mutant was already significantly diminished at lower concentration (300 μM) of AraC, while the P253G mutant started to show alterations in the response at 500 μM of AraC (Fig. 4C, D), suggesting that the Cys 256 is crucial for AraC/p75^NTR^ TMD interaction. Taken together, these data demonstrate that Pro 253 and Cys 256 are required for the AraC /p75^NTR^ TMD interaction.

**Fig. 5:**
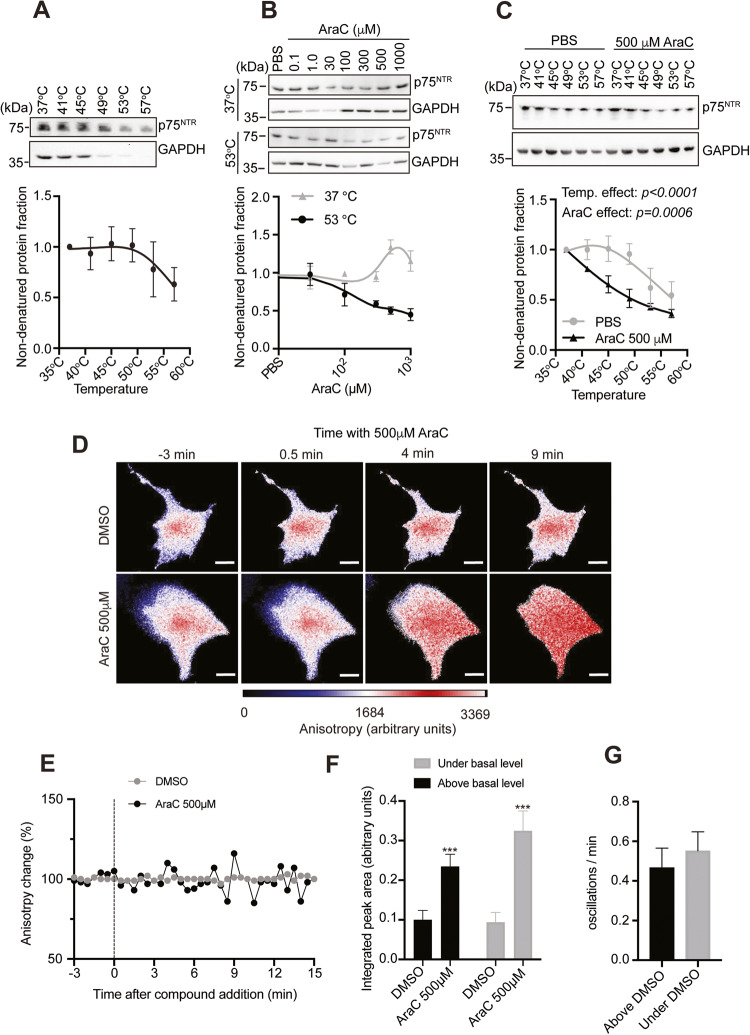
AraC changes the properties of the p75^NTR^ protein. **A** Representative immunoblot probed for p75^NTR^ and GAPDH and quantification of CETSA for p75^NTR^ protein response to different temperatures. **B** Representative immunoblot probed for p75^NTR^ and GAPDH and quantification of p75^NTR^ CETSA for AraC dose–response on protein lysate extracted from HEK293 cells constitutively overexpressing p75^NTR^. The lysate was heated to 37 °C and 53 °C. **C** Representative immunoblot probed for p75^NTR^ and GAPDH and quantification of p75^NTR^ CETSA for p75^NTR^ response to temperature after treatment with either PBS (vehicle) or 500 μM AraC. Mean ± s.e.m. of data from 3 to 6 separate cultures is shown for p75^NTR^. **D** Live cell homo-FRET anisotropy of p75^NTR^ in COS-7 cells in response to AraC. Shown are representative time-lapse images before (−3 min) and after (0.5, 4 and 9 min) addition of 500 µM AraC or DMSO (control). Scale bars, 5 μm. **E** Live cell homo-FRET anisotropy of p75^NTR^ in COS-7 cells in response to AraC. Shown are representative traces of average anisotropy change after the addition of AraC (500 µM at 0 min) or vehicle in cells expressing wild-type rat p75^NTR^. **F** Integrated peak area of live cell homo-FRET anisotropy of p75^NTR^ in COS-7 cells in response to AraC (considering the area under and above *y* = 1). Results are plotted as means ± SD (*N* = 7). ***P < 0.001; Student *t*-test. **G** Oscillations/min of live cell homo-FRET anisotropy of p75^NTR^ in COS7 cells in response to AraC (considering the vehicle oscillations as threshold). Results are plotted as means ± SD (*N* = 7).

We then sought to test whether AraC could interact with full-length p75^NTR^ protein. To this end, we used the CETSA which monitors protein–drug interaction by assessing ligand-induced changes in the thermal stability of the protein of interest [55]. First, using protein lysate from 293 T HEK cells that have constitutive expression of p75^NTR^, we generated the p75^NTR^ melting curve and found that p75^NTR^ starts to melt at 53 °C (Fig. 5A). Then, using the lysate from p75^NTR^ expressing HEK cells, we performed isothermal dose response (ITDR)-CETSA to assess the p75^NTR^ protein thermal stability at 37 °C and 53 °C after addition of different concentrations of AraC. While the addition of AraC shifted the thermal stability of p75^NTR^ in general, the higher concentrations (100–1000 μM) of AraC led to a marked increase in p75^NTR^ destabilization at 53 °C (Fig. 5B), suggesting that AraC binding to p75^NTR^ result in thermal instability of the protein. Moreover, we found that the addition of 500 μM AraC led to p75^NTR^ denaturation at lower temperatures starting at 40 °C compared to the control group (Fig. 5C), further supporting AraC-mediated p75^NTR^ destabilization. Altogether, these data suggest that AraC interacts with full-length mammalian p75^NTR^.

Next, we sought to confirm that AraC could still bind to p75^NTR^ in intact cells using a homo-FRET anisotropy assay. COS-7 cells were transfected with a plasmid expressing a full-length rat p75^NTR^- EGFP* fusion [42], and 24 h later, the changes in anisotropy levels after treatment with DMSO (control) or AraC were recorded over a certain amount of time. Treatment with AraC induced oscillation of p75^NTR^ anisotropy at the cell membrane (Fig. 5D, E), resulting in a positive net change of the integrated peak area over 15 min treatment compared with vehicle (Fig. 5D, F). Interestingly, the frequency of the anisotropy oscillation after AraC treatment was similar to the anisotropy changes above or under the control basal line (DMSO average anisotropy values) (Fig. 5G). Together, these data demonstrate that AraC interacts with p75^NTR^.

### AraC selectively uncouples p75^NTR^ from the NFκB signalling pathway

**Fig. 6:**
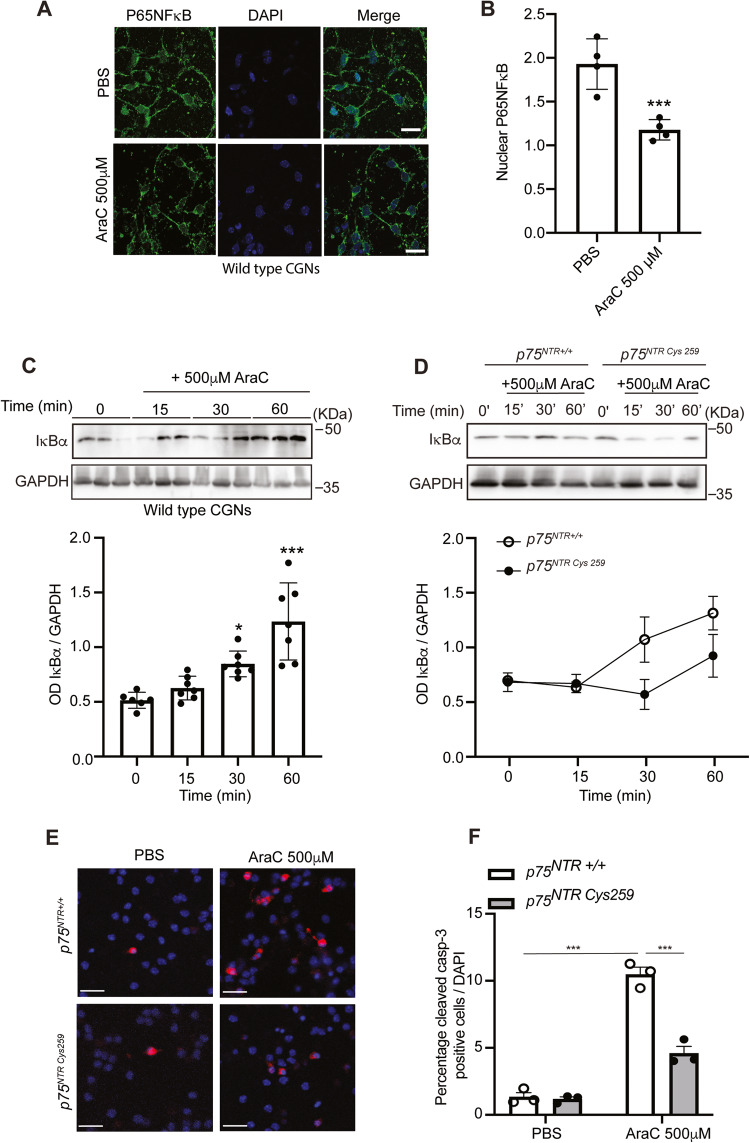
AraC selectively uncouples p75^NTR^ from NFκB signalling pathway. **A** Representative images of wild-type P7 CGNs cultured for 4 days, treated with either PBS (control) or 500 μM AraC for 60 min, fixed, stained for P65NFκB (green) and counterstained with DAPI (blue). Scale bar, 50 μM. **B** Quantification of the P65NFκB nuclear translocation in PBS (control) or neurons treated with 500 μM AraC for 60 min. Mean ± s.e.m. of data from four separate cultures, ***P < 0.001 compared to control, unpaired Student *t*-test) is shown. **C** Representative western blots probed with IκBα and GAPDH of lysate of wild-type P7 CGNs grown for 4 days prior to stimulation with 500 μM AraC for 15, 30 or 60 min. **D** Quantification of IκBα degradation in the total lysate of untreated wild-type P7 CGNs or neurons treated with 500 μM AraC for 15, 30 or 60 min. Mean ± sem of densitometry from 6 experiments (***P < 0.001; one-way ANOVA followed by Bonferroni test) is shown. **E** Representative images of wild type or C259A mutant P7 CGNs cultured for 4 DIV that were either treated with PBS (control) or 500 μM AraC for 24 h and stained for cleaved caspase 3 (red) and counterstained with DAPI (blue). Scale bars, 50 μm. **F** Quantification of percentage cleaved caspase 3 positive neurons wild type or C259A mutant CGNs treated with either PBS (control) or AraC (500 μM) for 24 h (total of 60 images per condition were counted). Mean ± s.e.m. of data from four separate cultures, ***P < 0.001 compared to control, one-way ANOVA followed by Bonferroni post hoc test) is shown.

We previously showed that activation of p75^NTR^-dependent nuclear factor kappa-light-chain-enhancer of activated B cells (NFκB) signalling pathway is crucial for the survival of CGNs [51]. Therefore, we evaluated whether AraC treatment induces uncoupling of p75^NTR^ from the NFκB signalling pathway. p75^NTR^-mediated activation of the NFκB pathway leads to the phosphorylation and subsequent degradation of IκBα (nuclear factor of kappa light polypeptide gene enhancer in B-cells inhibitor, alpha), which does not cover the nuclear localisation signal of the cytosolic P65NFκB anymore leading to its translocation to the nucleus [51, 56]. Neurons treated with AraC for 30 min at 4 DIV showed lower P65NFκB immunoreactivity in the nucleus compared to control (Fig. 6A, B), suggesting that P65NFκB remains bound to IκBα in the cytosol. Therefore, we evaluated the degradation of IκBα in untreated (naïve) cells and after treatment with 500 μM AraC. We observed a decrease in IκBα degradation after 30 and 60 min of AraC treatment (Fig. 6C). Since our AraC/p75^NTR^ data suggest that C259 is crucial for this interaction (Fig. 4D), we hypothesised that the effect of AraC on neurons lacking this residue will be abrogated. As expected, WT neurons treated with AraC had an accumulation of IκBα in the cytosol, while C256A neurons had a less pronounced IκBα accumulation after AraC treatment (Fig. 6D). Together, these data indicate that the AraC/p75^NTR^ TMD interaction leads to uncoupling of p75^NTR^ from the NFκB signalling pathway resulting in the accumulation of IkBα in the cytosol and less translocation of P65NFκB to the nucleus.

Finally, we evaluated apoptotic activity in wild-type and C259A neurons after 24 h treatment with AraC. As expected from our previous results, treatment of wild-type CGNs with AraC increased the percentage of cleaved caspase 3 positive cells in the cultures (Fig. 6E, F). On the other hand, although C259A CGNs treated with AraC also showed an increase in the number of cleaved caspase 3 positive neurons, the number of apoptotic cells was 50% lower than in the WT neurons (Fig. 6E, F). These results indicate that C259 is involved in t AraC/p75^NTR^ TMD interaction, but other residues are necessary since that mutation does not completely block the apoptotic effect induced by the drug.

### AraC/p75^NTR^ interaction does not affect p75^NTR^-dependent TrkB and RhoA signalling

Next, we dissected the signalling mechanism that AraC/p75^NTR^ employs to elicit cell death of mature CGNs. p75^NTR^ is known to engage several signalling pathways by interacting with other receptors and recruiting a number of adaptor proteins to its death domain, which lacks enzymatic activity [23, 50, 51, 56–60]. Moreover, p75^NTR^ has been suggested to increase the affinity of neurotrophins for Trk receptors [61–63]. To dissect the signalling mechanism that AraC/p75^NTR^ employs to elicit cell death of mature CGNs, we first evaluated whether the binding of AraC to p75^NTR^ TMD interferes with the activation of the TrkB signalling pathway that is indispensable for the survival of neurons [64]. Upon activation by neurotrophins or pharmacological drugs, TrkB is phosphorylated on several residues, including tyrosine 515 [64, 65]. Phosphorylation of tyrosine 515 regulates protein kinase B (AKT), which plays an important role in cell survival [66]. We, therefore, first evaluated the expression of TrkB in 4 DIV CGNs (Supplementary Fig. 2A) and the phosphorylation of TrkB, particularly on tyrosine 515, observing no changes upon AraC treatment (Supplementary Fig. 2C, D). This result suggests that the binding of AraC to p75^NTR^ TMD does not interfere with p75^NTR^-dependent TrkB activity. We then asked whether the AraC/p75^NTR^ interaction affects the signalling pathways (RhoA, JNK and NFκB pathways) that are downstream to p75^NTR^ [23, 60]. We assessed the AraC/p75^NTR^ interaction might modulate p75^NTR^-dependent RhoA activity in these neurons. However, CGNs treated at 4 DIV with AraC did not show any alteration in the levels of RhoA activity (Supplementary Fig. 2F).

### AraC/p75^NTR^- mediated inactivation of NFκB pathway exacerbates neurodegeneration by activating cell death/JNK pathway

**Fig. 7:**
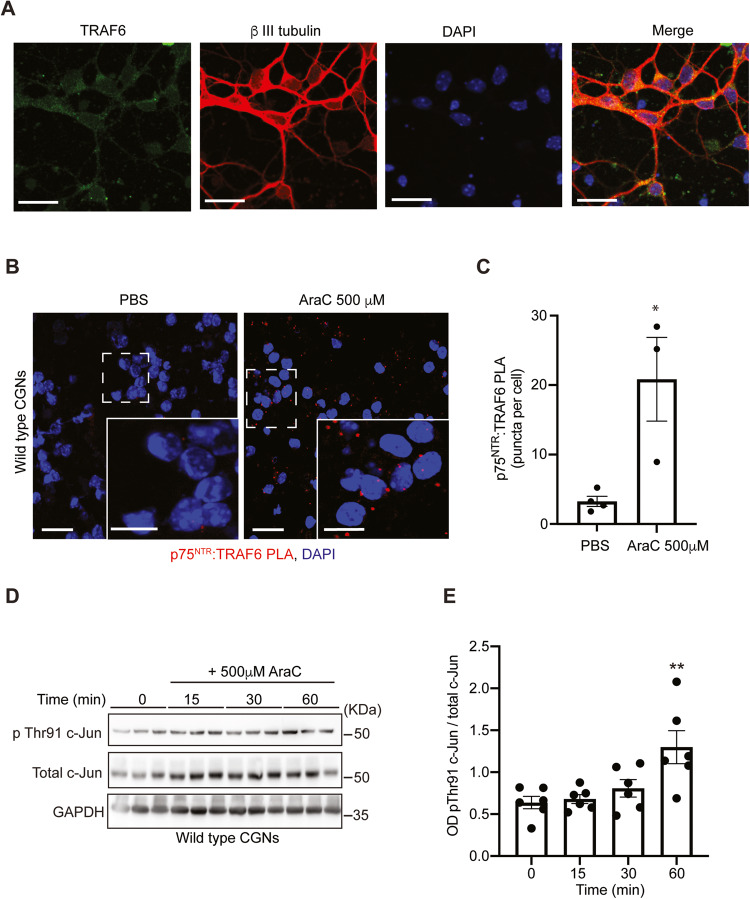
AraC/p75^NTR^- mediated inactivation of NFκB pathway exacerbates neuronal death by activating the JNK pathway. **A** Representative micrographs of wild-type P7 CGNs cultured for 4DIV and double stained with anti-TRAF6 together with anti- β III tubulin and counterstained with DAPI. Scale bars, 50 μm. **B** Micrographs of p75^NTR^:TRAF6 PLA (red) in CGNs treated with either PBS (control) or 500 μM AraC for 10 min. Images were selected from 25 images per condition from 3 to 4 separate experiments. Scale bar, 20 μm. **C** Quantification of p75^NTR^:TRAF6 PLA puncta in CGNs treated with either PBS (control) or 500 μM AraC for 10 min. Mean ± sem of data from 3 experiments (*P < 0.05; unpaired Student *t*-test) is shown. **D** Representative western blots probed with phospho-c-Jun (Thr91), total c-Jun and GAPDH of lysates of wild type P7 CGNs grown for 4 days prior to 15-, 30- or 60-min treatment with 500 μM AraC. **D** Quantification of c-Jun (Thr91) phosphorylation of total lysate of untreated wild-type P7 CGNs or neurons treated with 500 μM for 15, 30 and 60 min. Mean ± sem of densitometry from 4 experiments (*P < 0.05; one-way ANOVA followed by Bonferroni test) is shown.

We previously reported that uncoupling of p75^NTR^ from NFκB leads to cell death by activation of the JNK pathway [51]. In the absence of RIP2, an adaptor protein linking p75^NTR^ to the NFκB pathway, p75^NTR^ binds to TRAF6, another adaptor protein that links p75^NTR^ to the JNK apoptotic pathway. For this reason, we asked whether AraC-mediated inactivation of the NFκB pathway could lead to the activation of the JNK pathway, exacerbating neuronal apoptosis. First, we confirmed the expression of TRAF6 in 4 DIV CGNs (Fig. 7A). Next, the recruitment of TRAF6 to the intracellular domain of p75^NTR^ after AraC treatment was assessed in the CGNs cultures at 4 DIV by PLA. We detected an increase in p75^NTR^:TRAF6 PLA puncta (Fig. 7B, C) that suggested that AraC alters the conformation of p75^NTR^, favouring the binding of TRAF6. Then, we evaluated the activation of the JNK apoptotic pathway by assessing the phosphorylation of c-Jun on threonine 91 (Thr91), which has been linked to cell death in CGNs [67]. Indeed, the interaction of AraC with wild-type p75^NTR^ increased the phosphorylation of c-Jun on Threonine 91 residue (Fig. 7D, E). Altogether, these data indicate that treatment of CGNs with a high concentration of AraC inhibits the NFκB survival pathway and potentiates the JNK apoptotic pathway.

## Discussion

AraC has successfully been used as a chemotherapeutic for decades, being the most effective chemotherapy for the treatment of several cancers, including AML, acute lymphatic leukaemia (ALL) and non-Hodgkin’s lymphoma [1–4]. Similar to other chemotherapy treatments, AraC has several side effects, including neurotoxicity [3, 6, 7]. Interestingly, AraC-induced neurotoxicity is age-dependent; the older the patient is, the more severe the neurotoxicity, as older patients often require higher doses of AraC treatment [11]. In the current study, we show that high doses of AraC induce apoptosis in mature cerebellar neurons. These results are in agreement with observations made in adult cancer patients under the HIDAC treatment regime, who developed cerebellar atrophy and shrinkage leading to impaired cerebellar function that, in some cases, was permanent [11, 44]. As expected, the mature CGNs in vitro express the death receptor, p75^NTR^. Although it has been suggested that p75^NTR^ expression is markedly reduced in adult neurons [17], the expression of this receptor is not completely abrogated in the adult cerebellum [19, 51]. Moreover, recent single-cell RNA sequencing data confirms the expression of p75^NTR^ in the majority of adult cerebellar neurons [68]. Interestingly, acute leukaemia patients show expression of p75^NTR^ both on malignant and normal lymphocytes, as well as an increase in expression of p75^NTR^ in serum, bone marrow and peripheral blood cells [24, 25]. Moreover, our data show that AraC treatment increases the expression of p75^NTR^ in mature CGNs. Therefore, it is plausible that there is also an increase of p75^NTR^ in the cerebellum of leukaemia patients making them more susceptible to AraC-mediated neurodegeneration through p75^NTR^. In agreement with this, our data demonstrate that deletion of p75^NTR^ in mature CGNs prevented AraC-induced apoptosis.

P75^NTR^ facilitates different effects in CGNs, including axonal degeneration, cell survival, apoptosis and growth inhibition [22, 23]. These effects are achieved due to p75^NTR^’s capability to couple different signalling pathways, including NFκB, JNK/caspase and RhoDGI/RhoA pathways in these neurons [23, 60]. This raises the question as to why the interaction of AraC to p75^NTR^ induces neurite degeneration and cell death and not survival or growth inhibition in mature CGNs. It is noteworthy that the outcome of AraC interaction with p75^NTR^ depends on the availability of adaptor proteins (TRAF6, RIP2 and RhoA) and the signalling pathway that will be engaged.

We find that AraC binds to the TMD of p75^NTR^. Recent findings have demonstrated that a couple of small molecules bind to the TMD of certain receptors; for instance, SB394725 binds TMD of the thrombopoietin receptor [69], and NCS49652, a compound that binds to the TMD of p75^NTR^ [33]. The binding of AraC to p75^NTR^ TMD leads to anisotropy oscillations that are different to those induced by the binding of NSC49652 [33], suggesting that the two compounds bind to different regions of the p75^NTR^ TMD. In fact, while NSC496532 binds to Ile252, Pro253, Val 254, Cys256, and serine (Ser) 257 [33], our data suggest that Cys256 and Pro253 contribute to AraC/p75^NTR^ TMD interaction. We suggest that this specificity to these two residues could be the reason why AraC does not bind to other members of the TNF superfamily that p75^NTR^ belongs to [70, 71].

Interestingly, the interaction of AraC to Cys256 and Pro253 residues in p75^NTR^ TMD enables AraC to specifically uncouple p75^NTR^ from the NFκB pathway and not any other p75^NTR^- dependent pathway. We previously reported that uncoupling of p75^NTR^ from the NFκB pathway leads to apoptosis of CGNs during cerebellar development [51]. Moreover, we showed that in the absence of RIP2, p75^NTR^ recruits TRAF6, which couples the receptor to the JNK pathway, leading to an increase in the apoptotic activity of CGNs [51]. Although the wild-type neurons in the current study express both RIP2 and TRAF6, we speculated that the binding of AraC to p75^NTR^ TMD changes the conformation of p75^NTR^, hindering the recruitment of RIP2 to p75^NTR^ death domain. This new conformation favours the binding of TRAF6 to p75^NTR^, inducing the apoptotic activity of p75^NTR^ observed in these neurons. Indeed, our data show that treatment of mature CGNs with AraC leads to increased p75^NTR^:TRAF6 interaction rendering its specificity of activating the TRAF6/JNK cell death pathway.

Although the current work has focused on the effect of AraC on mature CGNs, we speculate that mature neurons in other brain regions that express p75^NTR^ could also be affected by this treatment. Interestingly, AraC-treated patients also exhibit somnolence and drowsiness [72, 73], reinforcing the notion that this drug could alter the function of other brain regions besides the cerebellum. Intriguingly, one brain region that expresses p75^NTR^ in abundance in the adult brain is the basal forebrain [18], which has been implicated in the homeostatic regulation of sleep [74–76]. It is, therefore, plausible that the somnolence and drowsiness phenotype observed in patients on HIDAC is a result of AraC/p75^NTR^-mediated neurite degeneration and cell death of the neurons in the basal forebrain.

**Fig. 8:**
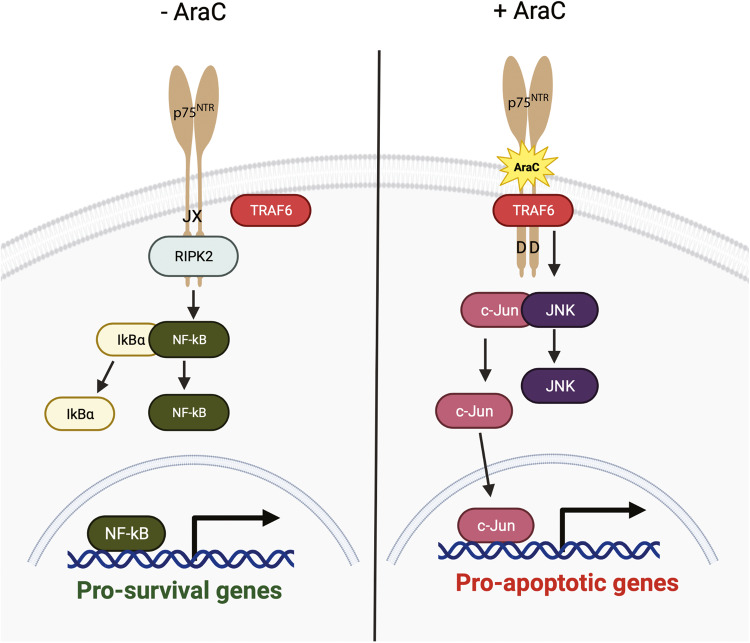
Molecular mechanism of AraC/p75^NTR^ interaction in the regulation of neuronal death. This schematic diagram depicts p75^NTR^ signalling in healthy mature neurons and when AraC interacts with P75^NTR^. Upon AraC binding to P75^NTR^, RIPK2 is dislodged from the death domain (DD), allowing the exposure of the juxta membrane (JX), where TRAF6 binds. The binding of TRAF6 to p75^NTR^ activates the JNK pathways that lead to the translocation of c-Jun to the nucleus and transcription of pro-apoptotic genes. Figure produced in BioRender.

In conclusion, our data show that p75^NTR^ facilitates AraC-induced cell death of mature CGNs by uncoupling p75^NTR^ from NFκB pathway and exacerbating cell death/JNK pathway, contributing to cerebellar degeneration (Fig. 8). Our data elucidates the molecular mechanisms of AraC-mediated neurite degeneration and cell death of mature neurons, providing a new molecular target for developing treatments to counteract the side effects of AraC in the CNS.

## Supplementary information

Supplementary figure legends

Supplementary Figure 1

Supplementary Figure 2

Full and uncropped western blots-Revised

Checklist

## Data Availability

All the data used and analysed for this study are available from the corresponding author upon reasonable request.
