# Supplementary material for: AraC interacts with p75NTR transmembrane domain to induce cell death of mature neurons
Source: Cell Death Dis. 2023 Jul 17;14(7):440. doi: 10.1038/s41419-023-05979-7 (PMC10352303; doi:10.1038/s41419-023-05979-7)
Supplement: Supplementary file 1 — Supplementary figure legends [file 41419_2023_5979_MOESM1_ESM.docx]

**Supplementary figure 1.** **No glial cells in culture treated with AraC.**

(**A-B**) Representative micrographs of wild type P7 CGNs cultured with or without AraC for 4 DIVand triple stained with anti- β III tubulin (neurons), anti-GFAP (astrocytes) and anti-Iba1 (microglia) and counterstained with DAPI. (**C**) Representative micrographs of wild type P7 CGNs cultured with AraC for 4 DIVand triple stained with anti-TAG1, anti- β III tubulin and anti-MAP2 and counterstained with DAPI. Scale bars, 50 μm.

**Supplementary figure 2. Binding of AraC to p75^NTR^ does not affect TrkB autophosphorylation or activation of RhoA pathway.**

(**A**) Representative micrographs of wild type P7 CGNs cultured for 4 DIV and double stained with anti-TrkB together with anti-β III tubulin and counterstained with DAPI. Scale bars, 50 μm. (**B**) Dose response of AraC in the AraTM assay of TrkB. Results are plotted as means ± SD (N = 3). (**C**) Representative western blots probed with phospho-TrkB (Y515), total TrkB and GAPDH of lysates of wild type P7 CGNs grown for 4 days prior to 15-, 30- or 60-minutes treatment with 500μM AraC. (**D-E**) Quantification of phosphorylation of the 140 KDa TrkB (**D**) and 90 KDa TrkB (**E**) in total lysate of untreated wildtype P7 CGNs or neurons treated with 500μM AraC for 15, 30 or 60 minutes. Mean ± sem of densitometry from 6 separate experiments is shown. (**F**) Analysis of RhoA-GTP levels in cerebellar extracts prepared from P7 wild type. Mean ± sem of data from 3 experiments is shown.
