# Supplementary figures and images for: AraC interacts with p75NTR transmembrane domain to induce cell death of mature neurons
Source: Cell Death Dis. 2023 Jul 17;14(7):440. doi: 10.1038/s41419-023-05979-7 (PMC10352303; doi:10.1038/s41419-023-05979-7)

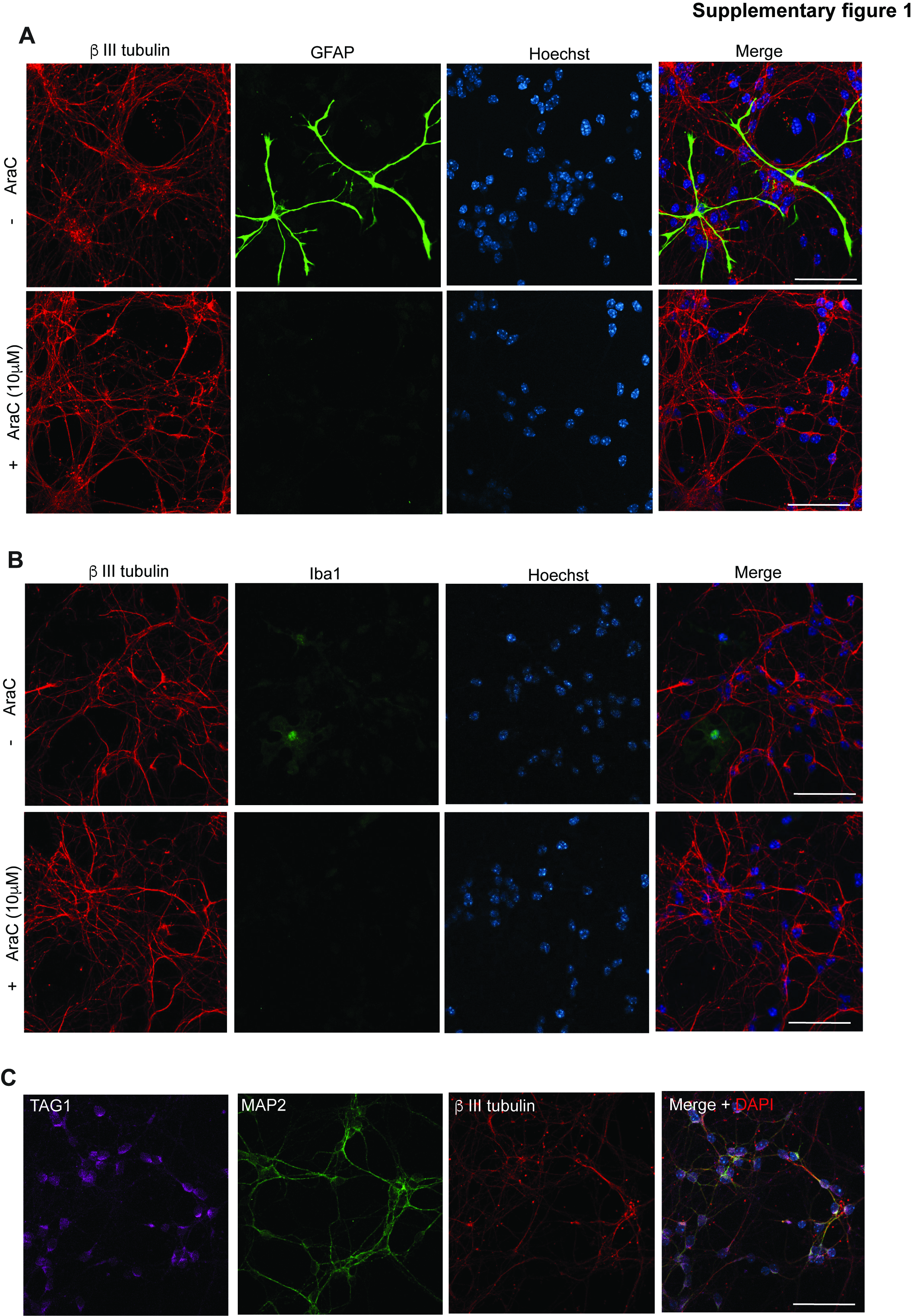

Supplement: Supplementary file 2 — Supplementary Figure 1 [file 41419_2023_5979_MOESM2_ESM.tif]

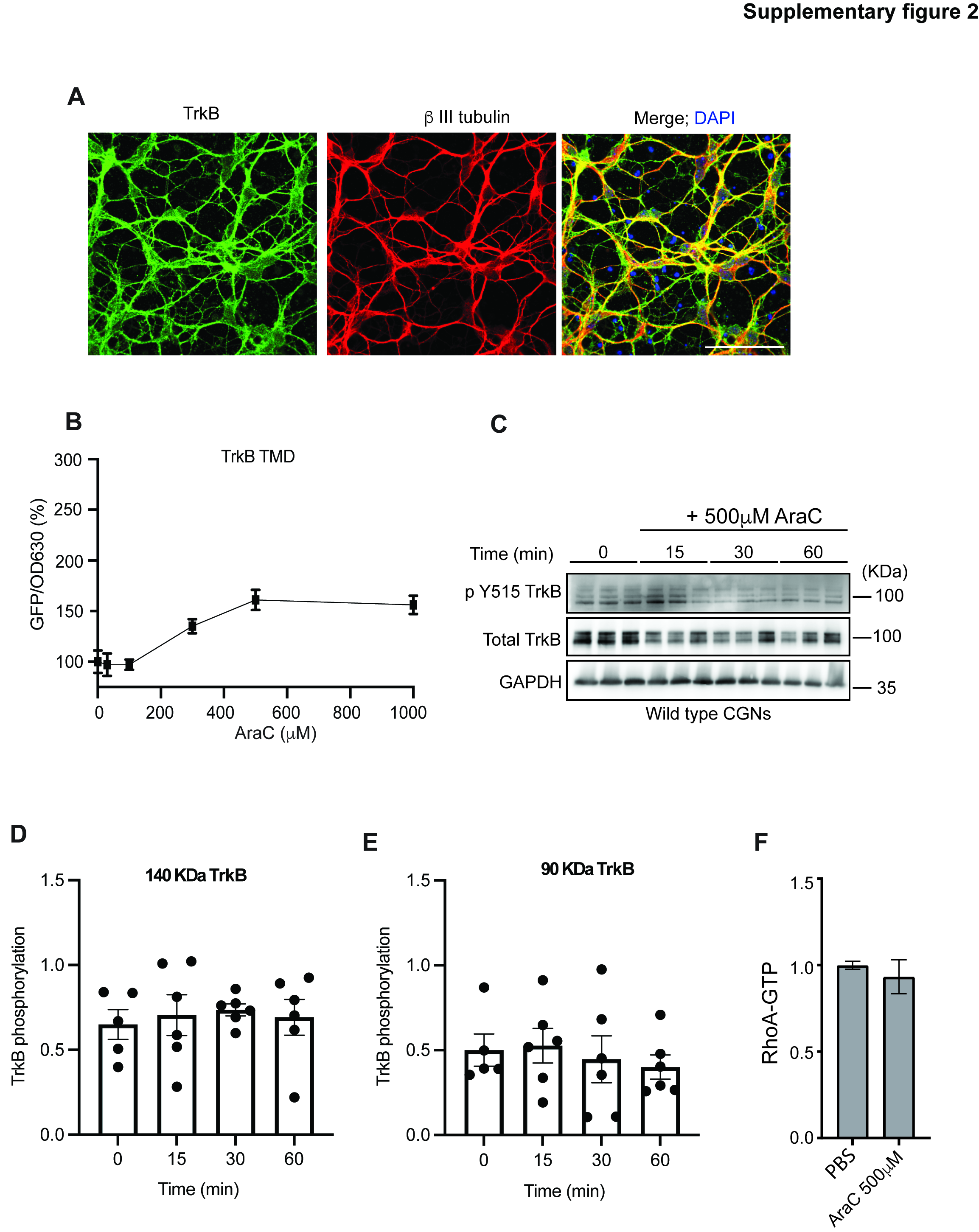

Supplement: Supplementary file 3 — Supplementary Figure 2 [file 41419_2023_5979_MOESM3_ESM.tif]

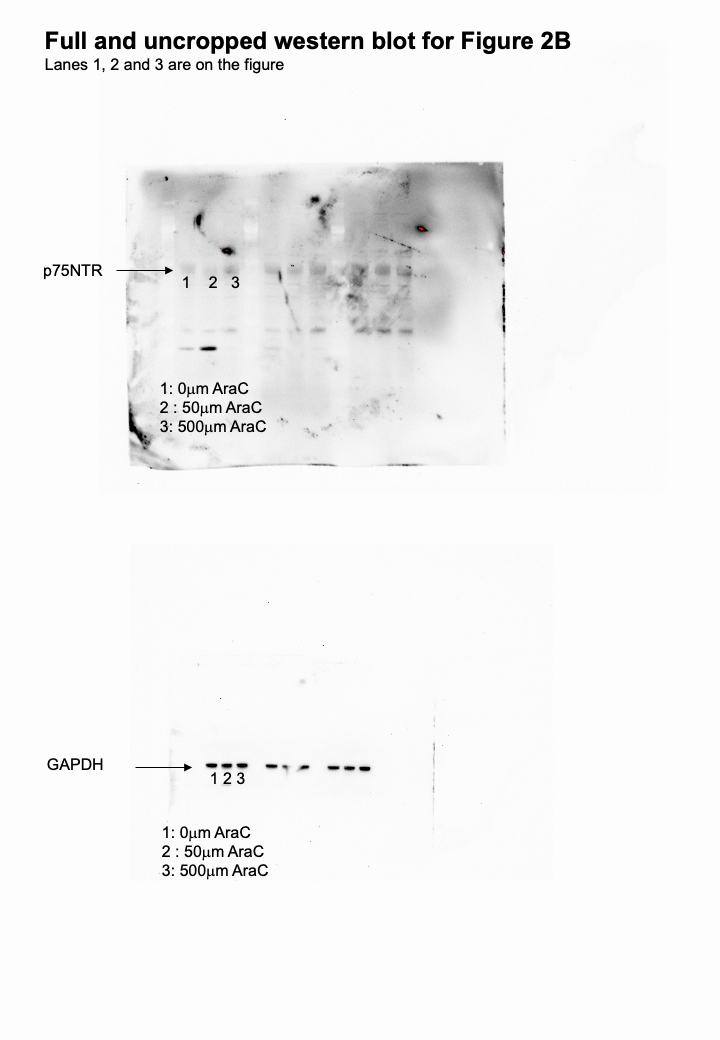

Supplement: Supplementary file 4 — Full and uncropped western blots-Revised [file 41419_2023_5979_MOESM4_ESM.tif]
